# Impact of COVID-19 on Mental Health of Chinese Residents in Its Initial Stage

**DOI:** 10.3389/fpsyg.2021.722093

**Published:** 2021-12-16

**Authors:** Linchuan Yang, Yunhong Liu, Li Han, Yibin Ao, Hongtai Yang

**Affiliations:** ^1^Department of Urban and Rural Planning, School of Architecture, Southwest Jiaotong University, Chengdu, China; ^2^College of Environment and Civil Engineering, Chengdu University of Technology, Chengdu, China; ^3^School of Transportation and Logistics, Southwest Jiaotong University, Chengdu, China

**Keywords:** impact of event scale-revised, public anxiety, state-trait anxiety inventory, COVID-19, post-traumatic stress disorder, questionnaire survey, China

## Abstract

This study aims to investigate the effect of coronavirus disease 2019 (COVID-19) on the Chinese public’s mental health during its early stage. We collected the data through an online questionnaire survey. Specifically, we adopted the impact of event scale-revised (IES-R) and state-trait anxiety inventory (STAI) to assess symptomatic responses to exposure to traumatic life events and public anxiety, respectively, in the COVID-19 pandemic outbreak. Then, we evaluated the differences in the scores among various socio-demographic groups using Kruskal-Wakkis H tests and *t*-tests and analyzed the IES-R, state anxiety (SA) score, and trait anxiety (TA) score using the Pearson correlation analysis. Finally, we conducted a path analysis to determine the mediating role of post-traumatic stress disorder symptoms (measured by the IES-R) in the relationship between TA and SA. The results show that the average of the SA and TA scores were 48.0 ± 10.4 and 38.0 ± 8.2, respectively; the respondents who suffered from mild, moderate, and severe psychological impacts because of the health crisis accounted for 21.9, 5.2, and 13.1%, respectively; farmers have the highest IES-R score than others; people with the highest income have the lowest SA level; a significant positive correlation existed between the IES-R and STAI scores; and TA produces both direct and indirect (through the IES-R) effects on SA. Overall, the general Chinese public exhibited much higher anxiety levels than normal in the early days of the pandemic outbreak. Accordingly, we strongly recommend psychological counseling and intervention support to mitigate the adverse psychological impacts of such an event.

## Introduction

Since the 1950s, the number effect of natural and human-related disasters has considerably increased (Li et al., 2020, 2021; Ao et al., 2021). There have been many epidemics in recent decades. On March 12, 2003, the WHO issued an unprecedented global alert. In particular, it declared an outbreak of severe acute respiratory syndrome (SARS), which became the first severe human-to-human disease in the 21st century (Wu et al., 2005). The virus spread rapidly in Asia, causing over 8,000 confirmed cases and at least 774 deaths. Its inherent scientific uncertainty also led to epidemic fear and suffering in various communities (Cheng and Wong, 2005). Similarly, in 2012, the Middle East respiratory syndrome (MERS) coronavirus emerged as a devastating public health threat (Khan et al., 2017). The epidemic caused human panic and financial loss and affected the mental health of infectious disease survivors and medical workers.

At the end of December 2019, Wuhan, the capital of Hubei Province (China) witnessed many cases of unknown viral pneumonia. On January 7, 2020, the Chinese Center for Disease Control and Prevention identified and isolated a new coronavirus disease. The novel coronavirus was named SARS-CoV-2. SARS-CoV-2 can spread through close contact between people. The novel coronavirus disease 2019 (COVID-19) outbreak spread rapidly and affected all regions of China and many other countries of the world. On January 30, 2020, the WHO Emergency Committee announced that the COVID-19 outbreak had become a public health emergency of international concern (Lee, 2020). In March 2020, the deadly virus swiftly swept across the whole world. Surely, the rapid spread of the pandemic has highly affected the physical and mental health of the global community. Therefore, the mental health of the public during this health crisis warrants scholarly and medical attention.

Public health emergencies may adversely affect the mental health of the public (Di Crosta et al., 2020, 2021; Cannito et al., 2021; Chirumbolo et al., 2021). This effect can manifest into different results, such as burnout, post-traumatic stress disorder (PTSD, a recurrent mental and physical distress happening after dangerous or catastrophic, transient, or long-lasting situations), anxiety, and depressive symptoms (Lancee et al., 2008; Fontalba-Navas et al., 2017; Probst et al., 2020; Xu et al., 2020; Daniali and Flaten, 2021; Wheaton et al., 2021). Previous studies consistently found that PTSD increases the prevalence of infectious diseases among survivors (Ro et al., 2017). Therefore, the timely detection of the symptoms of patients and psychological intervention will help reduce the psychological impact of health crises on the public and consequentially benefit the whole society.

To understand the mental health status of the Chinese public in the early COVID-19 stage, this study used the impact of event scale-revised (IES-R) and the state-trait anxiety inventory (STAI) to collect data through an online questionnaire survey. These two scales determine the extent of the COVID-19 effect on the public’s mental health during the tumultuous time. On the basis of our collected data, we analyze the relationship between the IES-R, SA, and TA scores of the public to provide theoretical support for their subsequent psychological intervention, treatment, and recovery. Overall, this study aims to provide a valuable basis for individuals, government agencies, and medical personnel to take timely and effective measures to alleviate the psychological anxiety of the public developed in the COVID-19 outbreak. We also hope that these measures will reduce the lasting psychological adverse effects caused by such a health crisis.

The contributions of this paper include (1) examining the effect of COVID-19 on mental health during the early days of the pandemic; (2) comparing the COVID-19 effect on people with different socio-demographic characteristics; (3) examining the association of the IES-R, SA, and TA scores; and (4) determining that the relationship between TA and SA is mediated by symptomatic responses to exposure to traumatic life events (measured by the IES-R), which contributes to a better understanding of the connection between the IES-R, SA, and TA scores.

The remainder of this paper is organized as follows. Section “Materials and Methods” introduces the data collected for analysis. Section “Result” presents the empirical results. Section “Discussion” discusses the findings of this study. The final section (Section “Concluding Remarks”) winds up the paper and identifies avenues for further research.

## Materials and Methods

### Measures

#### Impact of Event Scale-Revised

The IES-R, originally proposed by Weiss and Marmar (1997), is a self-report measure to evaluate subjective distress caused by traumatic events. IES-R respondents are required to determine a certain stressful life event and demonstrate the magnitude of the bother in the past week (measured by Likert-type scores). The IES-R is one of the most popular measures of subjective distress assessment around the world. It has been translated into many languages for international use. Its internal consistency and test-retest reliability have been extensively confirmed in numerous studies (Weiss, 2007; Ripon et al., 2020; Wang et al., 2020).

The IES-R is the enhanced version of the Impact of Event Scale (IES), a simple yet compelling self-report measure initially put forward by Horowitz et al. (1979). The original IES, which has been widely employed to measure PTSD symptoms among various populations, comprises 15 items for the diagnosis of PTSD and 2 subscales, namely, intrusion (7 items) and avoidance (8 items). The IES-R adds 7 items to the original IES and creates a new subscale, namely, hyperarousal. As such, it contains a total of 22 items (= 7 + 8 + 7) and 3 subscales, namely, intrusion, avoidance, and hyperarousal (Wagner and Waters, 2014; Zhang et al., 2014).

The scoring method of the IES-R for each item is 0 = Not at all, 1 = A little bit, 2 = Moderately, 3 = Quite a bit, and 4 = Extremely, which is slightly from that of the IES (0, 1, 3, 5). Therefore, the total IES-R score ranges from 0 to 88 (= 22 × 4). According to the IES-R score, people can usually be categorized into four groups: 0–23 (normal), 24–32 (mild psychological impact), 33–36 (moderate psychological impact), and ≥ 37 (severe psychological impact; Alkhamees et al., 2020).

Because the COVID-19 pandemic was still ongoing at the period of data collection, Item 1 (“Any reminder brought back feelings about it”) and Item 12 (“I was aware that I still had a lot of feelings about it, but I didn’t deal with them”) were excluded in this study. Therefore, in this study, a total of 20 IES-R items are used, and the maximum IES-R score is 80. We slightly modified the cut-off values according to the maximum score comparison (80 for the revised version vs. 88 for the original) and thus categorized people into four groups: 0–20 (normal), 21–29 (mild psychological impact), 30–33 (moderate psychological impact), and ≥ 34 (severe psychological impact).

#### State-Trait Anxiety Inventory

This study uses the STAI to evaluate the state and trait anxieties of the public. The STAI is a self-reporting tool used to assess the level of anxiety associated with a situation and is made up of the state anxiety (SA) inventory and the trait anxiety (TA) inventory. A higher score means a higher anxiety level (Dennis et al., 2013).

The SA scale is used to assess the current state of anxiety. It measures how respondents feel “now,” using items that measure subjective feelings, namely, fear, tension, worry, and autonomic nervous system activation/arousal. By contrast, the TA scale is used to evaluate relatively stable aspects of “anxiety propensity,” including general states of calm, confidence, and security (Julian, 2011). The STAI has a total of 40 items, including 20 SA ones and 20 TA ones. For SA, 10 items capture negative emotions, and the other 10 describe positive emotions. For TA, 11 items capture negative emotions, and the other nine describe positive emotions.

The STAI uses a 4-point Likert-type score (ranging from 1 to 4) for all 40 items. Therefore, the SA and TA scores are 20–80. According to their STAI score, people can be categorized into four groups: no anxiety (= 20), mild anxiety (21–39), moderate anxiety (40–59), and severe anxiety (60–80; Liu et al., 2020).

### Data

Many socio-demographic factors are found to affect the transmission and spread of infectious diseases, such as influenza (Liu, 2020). Recently, research on the influence of COVID-19 shows that gender is a consistent predictor of psychological outcomes. Specifically, females exhibited a higher level of psychological distress than males, indicating moderate levels of anxiety. Concerning age groups, although the results subtly varied across studies, the young (18–30 years old) and older adults (≥ 60 years old) commonly reported the highest level of psychological distress (Mazza et al., 2020). Moreover, the poor, the disabled, the marginalized, those on insecure employment, and other vulnerable groups had the greatest risk of infection and indirect consequences (Lee and Morling, 2020). Therefore, in addition to IES-R and STAI scores, our survey records many socio-demographic factors, such as gender, marriage, occupation, education attainment, annual household income, and recent location.

A pilot survey was conducted on February 8, 2020. We meticulously revised the questionnaire according to the feedback from the pilot survey. We distributed its final version on February 10, 2020, through an online questionnaire survey platform (“Questionnaire Star,” Changsha Ranxing Information Technology Co., Ltd., Changsha, China). Until 00:00 on February 19, we collected 956 questionnaires. After eliminating 200 invalid questionnaires (e.g., uncompleted questionnaires and respondents aged <17 years), we retained 736 valid ones for subsequent analyses (effective rate = 76.98%). The respondents covered a wide range of locations: 33 provincial administrative regions in China. More information regarding this online survey can be found in our previous publication (Ao et al., 2020). Table 1 shows the demographic information of the 736 respondents.

**Table 1:** Demographic information of the respondents.

Variable	Number of observations	Percentage (%)	Variable	Number of observations	Percentage (%)
*Gender*			*Education attainment*		
Male	307	41.7	Middle school or below	30	4.1
Female	429	58.3	High school/technical secondary school/vocational high school	39	5.3
*Age (years)*			Junior college	104	14.1
Under 25	224	30.4	Undergraduate	299	40.6
26–30	141	19.2	Master	170	23.1
31–40	223	30.3	Doctor	94	12.8
41–50	113	15.4	*Annual household income (RMB)*		
51–60	31	4.2	Under 30,000	61	8.3
Above 60	4	0.5	30,000–50,000	92	12.5
*Marital status*			60,000–100,000	164	22.3
Unmarried	315	42.8	110,000–150,000	122	16.6
Married	406	55.2	160,000–200,000	100	13.6
Divorced	12	1.6	210,000–250,000	59	8
Widowed	3	0.4	260,000–300,000	52	7.1
*Occupation*			Over 300,000	86	11.7
Ordinary/enterprise employee	238	32.3	*Residential location*		
Government/public institution servant	215	29.2	Rural	122	16.6
Farmer	9	1.2	Township	136	18.5
College student	190	25.8	City	478	64.9
Others	84	11.4			

### Methods

We employed the SPSS 24.0 statistical software for the following statistical analysis: First, we determined the Cronbach’s α and KMO to verify the internal consistency and structural validity of the IES-R and STAI scores. Next, we sorted out the obtained socio-demographic information by frequency and descriptive statistics. Then, we used Kruskal-Wakkis H tests and *t*-tests to identify the score differences among different socio-demographic groups. Finally, we investigated the correlations among IES-R, SA, and TA through a correlation analysis and determined that TA affects SA both directly and indirectly (through IES-R, acting as mediator variable) using a path analysis (also known as causal modeling, latent variable models, and analysis of covariance structures).

## Results

The Cronbach’s α values of the IES-R, SA, and TA were determined to be 0.916, 0.919, and 0.862, respectively. The KMO values of the IES-R, SA, and TA were 0.943, 0.933, and 0.921, respectively. Therefore, we can conclude that our questionnaire data have excellent internal consistency and structural validity.

### IES-R Score Analysis

Table 2 shows the IES-R scores of the respondents. The IES-R score for all respondents was 18.8 ± 12.4. Those with scores above and below the mean accounted for 48 and 52%, respectively.

**Table 2:** IES-R score of the respondents Source: Ao et al. (2020).

Characteristic	Category	Mean	Standard Deviation
All respondents		18.8	12.4
Gender	Male	19.4	12.6
	Female	18.4	12.2
	*t*-statistic	−1.077	
	Value of *p*	0.281	
Age (years)	18–25	17.6	12.7
	26–30	19.4	12.3
	31–40	19.4	12.0
	41–50	18.6	12.9
	51–60	22.2	11.0
	61–70	15.8	13.6
	*H*-statistic	8.95	
	Value of *p*	0.176	
Marital status	Unmarried	19.0	13.5
	Married	18.2	11.5
	Divorced	20.6	12.4
	Widowed	13.0	5.0
	*H*-statistic	1.258	
	Value of *p*	0.739	
Current location	Rural	18.3	11.1
	Town	18.7	12.5
	City	19.0	12.7
	*H*-statistic	0.052	
	Value of *p*	0.974	
Education attainment	Junior secondary or below	18.8	12.5
	High school	21.9	13.6
	Junior college	19.5	10.9
	Undergraduate	18.1	12.5
	Master	19.9	12.3
	Doctor	17.2	13.0
	*H*-statistic	9.181	
	Value of *p*	0.102	
Annual household income (RMB)	Under 30,000	18.6	13.8
	30,000–50,000	21.1	13.2
	60,000–100,000	19.3	13.1
	110,000–150,000	18.1	11.2
	160,000–200,000	17.9	12.6
	210,000–250,000	19.1	11.2
	260,000–300,000	20.9	12.0
	Over 300,000	16.5	10.9
	*H*-statistic	9.508	
	Value of *p*	0.218	
Occupation	Ordinary employee/enterprise employee	18.8	11.7
	Government/public institution servant	19.4	12.3
	Farmer	28.9	8.5
	College student	17.8	12.1
	Others	18.9	13.1
	*H*-statistic	10.553	
	Value of *p*	0.032	

The IES-R score was 19.4 ± 12.6 for males and 18.4 ± 12.2 for females. Males and females were found to only have minor differences. Although previous empirical studies (Pyari et al., 2012) have identified that females are more likely to suffer from PTSD, we do not find consistent outcomes. Future studies with larger sample sizes are, therefore, needed to support or refute our results. In addition, people aged 51–60 years had the highest score (22.2 ± 11.0). Regarding marital status, divorced people had the highest score (20.6 ± 12.4). Furthermore, people with the highest academic degree (doctoral degree) had the lowest score (17.2 ± 13.0). Last but not least, among the occupation groups, farmers scored the highest (28.9 ± 8.5).

The empirical data also showed that the proportion of people who were not affected by the pandemic outbreak (IES-R score ≤ 20) was 59.9% (441 people). The proportion of people with mild (21 ≤ IES-R score ≤ 29), moderate (30 ≤ IES-R score ≤ 33), and severe (IES-R score ≥ 34) psychological impacts was 21.9% (161 people), 5.2% (38 people), and 13.1% (96 people), respectively. The results showed that the number of people affected by the pandemic outbreak in its early stage accounted for 40.1%. This result indicated that the pandemic had substantially affected the mental health of the masses.

### STAI Score Analysis

Table 3 reveals the SA and TA scores of the respondents. The average SA score of the respondents was 48.0 ± 10.4, and the average TA score was 38.0 ± 8.2. The SA score of the Chinese public affected by the health crisis was significantly higher than the individual TA score (*t* = 22.66, *p* < 0.01), and more than 79.6% of the respondents have a high degree of anxiety. This observation reveals that COVID-19 considerably affects the anxiety level of the respondents at its initial stage (the data collection period). Moreover, the detection rates of without anxiety, mild anxiety, moderate anxiety, and severe anxiety symptoms were 0.5, 19.8, 68.5, and 11.1%, respectively (Ao et al., 2020).

**Table 3:** STAI scores of the respondents.

Characteristic	Category	SA		TA	
		Mean	Standard Deviation	Mean	Standard Deviation
Gender	Male	48.6	10.6	38.0	7.7
	Female	47.5	10.2	38.0	8.5
	*t*-statistic	−1.152		−0.219	
	Value of *p*	0.249		0.826	
Age (years)	18–25	47.3	11.2	37.9	8.1
	26–30	48.9	10.5	37.5	8.1
	31–40	48.2	9.6	37.8	8.1
	41–50	47.6	9.6	38.7	8.5
	51–60	49.6	11.0	39.3	9.1
	61–70	50.0	9.5	42.5	7.0
	*H*-statistic	5.848		3.338	
	Value of *p*	0.440		0.765	
Marital status	Unmarried	48.0	11.1	38.1	8.4
	Married	48.0	9.80	37.9	8.0
	Divorced	49.3	9.8	39.6	9.2
	Widowed	40.0	5.0	42.7	6.8
	*H*-statistic	3.144		1.863	
	Value of *p*	0.37		0.601	
Current location	Rural	47.1	9.8	37.3	7.4
	Town	48.5	10.9	38.3	9.1
	City	48.1	10.4	38.1	8.1
	*H*-statistic	1.622		1.084	
	Value of *p*	0.444		0.581	
Education attainment	Junior secondary or below	47.8	10.0	36.2	8.6
	High school	49.4	10.5	38.5	7.5
	Junior college	49.2	11.3	37.2	7.2
	Undergraduate	47.9	10.6	38.2	8.3
	Master	47.4	10.0	38.4	8.3
	Doctor	47.7	9.2	37.9	9.0
	*H*-statistic	1.817		2.706	
	Value of *p*	0.874		0.745	
Annual household income (RMB)	Under 30,000	48.7	11.2	38.0	8.1
	30,000–50,000	48.1	10.4	38.4	8.7
	60,000–100,000	48.1	10.9	36.7	8.5
	110,000–150,000	48.1	10.5	38.4	8.0
	160,000–200,000	49.1	9.9	38.5	7.2
	210,000–250,000	48.7	8.0	38.5	8.8
	260,000–300,000	50.8	10.6	38.2	8.7
	Over 300,000	43.9	9.5	38.4	7.6
	*H*-statistic	18.963		8.353	
	Value of *p*	0.008		0.303	
Occupation	Ordinary employee/enterprise employee	48.2	10.2	37.3	7.7
	Government/public institution servant	47.9	9.4	38.9	8.5
	Farmer	52.0	12.0	36.1	9.0
	College student	47.1	11.8	38.4	8.7
	Others	49.5	9.5	37.0	7.1
	*H*-statistic	4.998		5.877	
	Value of *p*	0.287		0.209	
All respondents		48.0	10.4	38.0	8.2

In the early stage of the COVID-19 pandemic outbreak, the SA level of the sampled Chinese residents (48.0 ± 10.4) was significantly higher than the national norm (39.7 ± 8.9; *t* = 16.513, *p* < 0.001). This outcome, indicating that the pandemic significantly affected the public’s mental health in its early stage, is as expected. More explanations for STAI scores can be found in Ao et al. (2020).

### Analysis of Group Differences

In Tables 2 and 3, we truly see some differences in the IES-R, SA, and TA scores. A question arises as: whether a socio-demographic group has significantly different scores from others? To answer this question, we conducted two-tailed *t*-tests to assess the statistical significance of the difference in the mean score.

Table 4 shows the results of selected socio-demographic group differences in the IES-R score. A significant difference existed in the IES-R score between those with doctoral and high school degrees (*p* = 0.039) and among those with annual family income above 300,000, 30,000–50,000 yuan (*p* = 0.013), and 260,000–300,000 yuan (*p* = 0.042). Besides, the effect of the COVID-19 outbreak on the farmers in its early stage was higher than that on other occupational groups, indicating that they were more vulnerable to the pandemic.

**Table 4:** Group differences in the IES-R score.

Variable	(A) Education	(B) Education	Mean difference (A–B)	Standard error	Value of *p*
IES-R	Doctor	Junior secondary and below	−1.684	2.589	0.515
		High school	−4.851^*^	2.352	0.039
		Junior college	−2.38	1.757	0.176
		Undergraduate	−0.951	1.46	0.515
		Master	−2.78	1.587	0.08
**Variable**	**(C) Annual household income (RMB)**	**(D) Annual household income (RMB)**	**Mean difference (C–D)**	**Standard error**	**Value of *p***
IES-R	Over 300,000	Under 30,000	−2.284	2.067	0.269
		30,000–50,000	−4.633^*^	1.852	0.013
		60,000–100,000	−2.839	1.644	0.085
		110,000–150,000	−1.645	1.738	0.344
		160,000–200,000	−1.397	1.816	0.442
		210,000–250,000	−2.631	2.087	0.208
		260,000–300,000	−4.412^*^	2.169	0.042
**Variable**	**(E) Occupation**	**(F) Occupation**	**Mean difference (E–F)**	**Standard error**	**Value of *p***
IES-R	Farmer	Ordinary employees/enterprise employees	10.112^*^	4.188	0.016
		Government/public institution servant	9.540^*^	4.196	0.023
		College student	11.068^*^	4.207	0.009
		Others	9.948^*^	4.325	0.022

* *The mean difference is significant at the 5% level*.

Table 3 has shown that people with different annual incomes had different SA levels (*H* = 18.963, *p* = 0.008) but similar TA levels (*H* = 8.353, *p* = 0.303). Therefore, we aim to compare the SA levels of the richest (annual household income >300,000 yuan) and others.

Table 5 shows the results of selected socio-demographic group differences in the SA score. It revealed that a significant difference existed in the SA level, and the SA level of the richest was significantly lower than that of others. There are many possible explanations for this interesting observation. First, the richest residents have more resources, so they are better prepared for the COVID-19 pandemic. In other words, not-so-rich residents (e.g., salesman, car/truck driver, hairdresser, take-away delivery man, small restaurant owner, and small shop owner) may have a limited income and feel helpless in the large-scale closure (for an unknown time) because many of them have house/car loans, children educational expenses, etc. Second, public anxiety increment could come from social isolation due to COVID-19 restrictions. The richest residents often have a strong social network, even in a pandemic context, which makes them exhibit a lower level of SA. Last, subjective expectation matters. Financial resources have a significant influence on how individuals view their future and how much the pandemic will negatively impact their lives. People who are concerned about their financial resources are more prone to fear that the pandemic will ruin their lives. Furthermore, there are other possible explanations for the obtained results. Future studies with rigorous research design can be conducted to investigate this issue to reach a more persuasive conclusion.

**Table 5:** Group differences in the SA score.

Variable	(I) Annual household income (RMB)	(J) Annual household income (RMB)	Mean difference (I–J)	Standard error	Value of *p*
SA	Over 300,000	Under 30,000	−4.800^*^	1.720	0.005
		30,000–50,000	−4.258^*^	1.541	0.006
		60,000–100,000	−4.213^*^	1.368	0.002
		110,000–150,000	−4.259^*^	1.447	0.003
		160,000–200,000	−5.188^*^	1.511	0.001
		210,000–250,000	−4.789^*^	1.737	0.006
		260,000–300,000	−6.878^*^	1.805	0

* *The mean difference is significant at the 5% level*.

### Correlation Analysis Between the IES-R, SA, and TA Scores

To explore the relationship between the magnitude of the effect of the pandemic outbreak and the psychological anxiety of the respondents in its early stage, Pearson correlation analyses were conducted to determine the correlation between the IES-R and STAI scores. The results (Table 6) show that a significant positive correlation existed between the IES-R and SA scores (*r* = 0.629, *p* < 0.01) and the SA and TA scores (*r* = 0.178, *p* < 0.01). However, the correlation between IES-R and SA scores is statistically insignificant (*r* = 0.065, *p* > 0.05). These findings are in agreement with our expectations. Besides, there are some correlations between IES-R and STAI scores.

**Table 6:** Correlation analysis between the IES-R and STAI.

	IES-R				STAI					
	Hyperarousal	Intrusion	Avoidance	Total IES-R score	PE (TA)	NE (TA)	TA score	PE (SA)	NE (SA)	SA score
Hyperarousal (sum of 7 items)	1									
Intrusion (sum of 6 items)	0.755^**^	1								
Avoidance (sum of 7 items)	0.687^**^	0.600^**^	1							
Total IES-R score (sum of 20 items)	0.924^**^	0.851^**^	0.880^**^	1						
PE (TA; sum of 9 items)	−0.072	−0.061	−0.100^**^	−0.090^*^	1					
NE (TA; sum of 11 items)	0.272^**^	0.212^**^	0.235^**^	0.274^**^	0.258^**^	1				
TA score (sum of 20 items)	0.078^*^	0.057	0.038	0.065	0.883^**^	0.682^**^	1			
PE (SA; sum of 10 items)	0.370^**^	0.353^**^	0.268^**^	0.368^**^	0.227^**^	0.059	0.201^**^	1		
NE (SA; sum of 10 items)	0.686^**^	0.576^**^	0.525^**^	0.673^**^	−0.077^*^	0.309^**^	0.092^*^	0.366^**^	1	
SA score (sum of 20 items)	0.638^**^	0.561^**^	0.479^**^	0.629^**^	0.092^*^	0.222^**^	0.178^**^	0.828^**^	0.825^**^	1

^**^and ^*^ mean that correlation is significant at the 1 and 5% levels, respectively. PE (TA), positive emotion of TA; NE (TA), negative emotion of TA; PE (SA), positive emotion of SA; and NE (SA), negative emotion of SA.

### Path Analysis Between the IES-R, SA, and TA Scores

**Figure 1:**
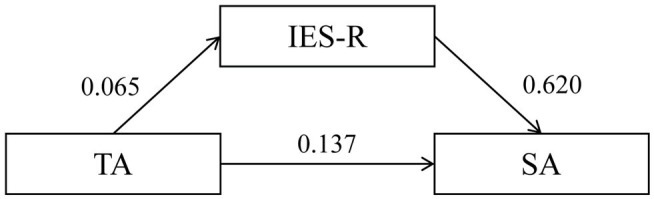
Standardized coefficients of variables in the path analysis.

The above correlation analysis has revealed the associations between the IES-R, SA, and TA scores. Following the logic of Ao et al. (2020), we can raise a question: is the relationship between TA and SA mediated by symptomatic responses to exposure to traumatic life events (as measured by IES-R)? A visual representation of the overall mediating relationship is presented in Figure 1.

A path analysis (Ceccato et al., 2021) was carried out to answer the above question (testing whether the mediating effect is significant or not) with the help of Software for Statistics and Data Science, STATA 16. Table 7 shows the results. It indicates that TA is fairly useful in predicting both IES-R and SA, and IES-R significantly affects SA. In other words, the path analysis outcomes demonstrate that the relationship between TA and SA is truly mediated by symptomatic responses to exposure to COVID-19.

**Table 7:** Path analysis results.

Variable	Unstandardized coefficient	Standard error	*t*-statistic	Standardized coefficient
**IES-R**				
TA	0.099^*^	0.06	1.77	0.065
Constant	15.095^**^	2.16	6.98	
**SA**				
IES-R	0.520^**^	0.02	21.88	0.620
TA	0.174^**^	0.04	4.84	0.137
Constant	31.634^**^	1.44	22.01	
Number of observations	736			

^**^and ^*^ mean that correlation is significant at the 1 and 10% levels, respectively.

## Discussion

Interestingly, government/public institution servants and college students have the lowest SA score, although their TA score is quite similar to that of people with other occupations. This may be because their statuses are more stable (e.g., free from unemployment and income reduction). Moreover, a significant difference was observed in the SA level among the richest and others.

In the early days of the COVID-19 outbreak, schools in all Chinese cities were required to close for an unknown time. The uncertainty of the return-to-school time and the potential negative effect on academic progress negatively affected students’ mental health (Wang et al., 2020). The respondents who had a high school degree scored the highest in the IES-R. During the outbreak, people were forced to stay at their homes because of a “stay at home” executive order imposed by the central government, which caused the public to have varying degrees of anxiety. Moreover, previous studies have shown that apart from the national health state, this pandemic has a major effect on the economies of nations and individuals (Cao et al., 2020).

Farmers scored the highest in the IES-R score. They are significantly different from other occupational groups, especially college students. A possible explanation is that compared with college students, farmers’ adaptability is weak. It is widely acknowledged that college students are the frontier group of new technologies and new ideas in society. They often have active thinking and can well use the Internet to obtain new information. Therefore, with exposure to new things, college students tend to accept information quickly and adapt well to changes in the environment. In addition, the farmers who participated in the survey were generally aged between 41 and 70 years. Many young people coming from rural areas have migrated to cities and towns, leading to an aging population in rural areas (Yang et al., 2021). The pandemic situation mostly affects farmers’ work and the agriculture industry. After the SARS epidemic, the Chinese government developed a population-based public health information system. At present, one can report directly to the government at the township level, resulting in quicker administrative responses (Li, 2011). On the basis of the above discussion, in the face of COVID-19, the township community could pay extra attention to the mental health problems reported by farmers and promptly seek help from the higher government in the occurrence of bigger problems.

PTSD is an anxiety disorder that often develops after a dangerous or disturbing event (Wang and Xu, 2016). The rapid spread of the virus has forced the public to stay at home. Brooks et al. (2020) indicated that people who had been isolated for more than 10 days showed significantly higher symptoms of post-traumatic stress than others. PTSD is typically correlated with hypertrophic neurosis, vigilance, and insomnia. Symptoms, such as anxiety and depression, can also occur. Drug abuse and suicidal thoughts are relatively common. The blockade in Wuhan has greatly slowed down the spread of COVID-19 and put people’s lives on pause, but it was short-lived. The continuous presence of the pandemic may have affected suspected individuals of PTSD. If psychological intervention is not promptly provided to patients with suspected PTSD, their psychological trauma may further deepen with extremely serious consequences.

The Pearson correlation analysis results showed that a significant positive correlation exists between the IES-R and SA scores. In addition, the path analysis outcomes revealed that the IES-R servers as a mediator in the relationship between the TA and SA scores. The Pearson correlation and path analysis outcomes jointly indicate that individuals who are easily affected by individual traits (low TA) will be more anxious under the influence of COVID-19 (low SA). As the development of COVID-19 affects more members of the public, the degree of psychological anxiety will increase, and its effect on low-TA people, high school students, and farmers will be more severe. Governments can pay more attention to the psychological state of these people.

## Concluding Remarks

This study explores the COVID-19 pandemic outbreak’s effect on the Chinese public’s mental health, especially anxiety, in its early stage based on the online survey instrument. It provides a research foundation for public psychological counseling and intervention. Policymakers and public mental health institutions can take effective measures to reduce public psychological anxiety during the pandemic and promote public psychological rehabilitation to help people restore their mental health more quickly after the pandemic.

On the basis of the analysis results, we recommend the following actions during the tumultuous time: (1) Public authorities should promptly establish psychological assistance platforms (e.g., online services and hotline consultation) with a wider reach and account for behavioral reactions of people (Briscese et al., 2020). Specifically, the government should support severely affected families and individuals to respond actively to the pandemic. (2) Government health institutions can improve rural public information service platforms to disseminate real-time updates regarding COVID-19 to farmers. (3) Schools can appropriately upgrade and calibrate preventive measures and emergency response capabilities and promote high school, undergraduate, and graduate students to return to school and continue to attend classes if deemed appropriate. (4) Public authorities can provide some subsidies to families experiencing severe income difficulties and allow the resumption of employment.

This study has the following shortcomings: First, this study has a small sample size and does not involve confirmed and cured patients. Therefore, it is impossible to know the extent of their exposure to COVID-19 events. This feature obviously has a serious effect on people’s mental health if they become ill or their family members (or close relatives) become ill and even die. Second, cross-sectional data were used in this study: the questionnaire survey was conducted in February 2020. At present, the pandemic has not completely disappeared. The pandemic has attacked in some Chinese cities, such as Beijing, Shanghai, Xiamen, Changsha, and Yangzhou. The struggle of China and other countries against the pandemic may constantly change the psychological impact of their residents. Therefore, tracking the mental health of Chinese (and people in other countries) is essential for making interesting discoveries.

## Data Availability Statement

The raw data supporting the conclusions of this article will be made available by the authors, without undue reservation.

## Ethics Statement

The studies involving human participants were reviewed and approved by the Chengdu University of Technology. The patients/participants provided their written informed consent to participate in this study.

## Author Contributions

LY: funding acquisition, validation, conceptualization, and writing – original draft. YL and LH: formal analysis, methodology, and writing – review and editing. YA: writing – review and editing, funding acquisition, validation, and supervision. HY: validation and writing – review and editing. All authors contributed to the article and approved the submitted version.

## Funding

This research was supported by the Fundamental Research Funds for the Central Universities of China (no. 2682021CX097) and 2020 Talent Cultivation Quality and Teaching Reform Project of Ideological and Political Theory Course, Chengdu University of Technology (no. 20800-2020SZ009).

## Conflict of Interest

The authors declare that the research was conducted in the absence of any commercial or financial relationships that could be construed as a potential conflict of interest.

## Publisher’s Note

All claims expressed in this article are solely those of the authors and do not necessarily represent those of their affiliated organizations, or those of the publisher, the editors and the reviewers. Any product that may be evaluated in this article, or claim that may be made by its manufacturer, is not guaranteed or endorsed by the publisher.
